# The effect of missing data on evolutionary analysis of sequence capture bycatch, with application to an agricultural pest

**DOI:** 10.1007/s00438-024-02097-7

**Published:** 2024-02-21

**Authors:** Leo A. Featherstone, Angela McGaughran

**Affiliations:** 1 Research School of Biology, Division of Ecology and Evolution, Australian National University, Canberra, ACT 2601 Australia; 2 Peter Doherty Institute for Infection and Immunity, The University of Melbourne, Melbourne, VIC 3000 Australia; 3 Te Aka Mātuatua, School of Science, University of Waikato, Private Bag 3105, Hamilton, 3240 New Zealand

**Keywords:** Bycatch, Evolutionary history, *Helicoverpa*, Mitogenomes, Targeted capture

## Abstract

Sequence capture is a genomic technique that selectively enriches target sequences before high throughput next-generation sequencing, to generate specific sequences of interest. Off-target or ‘bycatch’ data are often discarded from capture experiments, but can be leveraged to address evolutionary questions under some circumstances. Here, we investigated the effects of missing data on a variety of evolutionary analyses using bycatch from an exon capture experiment on the global pest moth, *Helicoverpa armigera*. We added > 200 new samples from across Australia in the form of mitogenomes obtained as bycatch from targeted sequence capture, and combined these into an additional larger dataset to total > 1000 mitochondrial cytochrome *c* oxidase subunit I (COI) sequences across the species’ global distribution. Using discriminant analysis of principal components and Bayesian coalescent analyses, we showed that mitogenomes assembled from bycatch with up to 75% missing data were able to return evolutionary inferences consistent with higher coverage datasets and the broader literature surrounding *H. armigera*. For example, low-coverage sequences broadly supported the delineation of two *H. armigera* subspecies and also provided new insights into the potential for geographic turnover among these subspecies. However, we also identified key effects of dataset coverage and composition on our results. Thus, low-coverage bycatch data can offer valuable information for population genetic and phylodynamic analyses, but caution is required to ensure the reduced information does not introduce confounding factors, such as sampling biases, that drive inference. We encourage more researchers to consider maximizing the potential of the targeted sequence approach by examining evolutionary questions with their off-target bycatch where possible—especially in cases where no previous mitochondrial data exists—but recommend stratifying data at different genome coverage thresholds to separate sampling effects from genuine genomic signals, and to understand their implications for evolutionary research.

**Supplementary Information:**

The online version contains supplementary material available at 10.1007/s00438-024-02097-7.

## Introduction

Targeted capture, in which selected regions of the genome are sequenced following enrichment from a whole genomic DNA extract, produces sequence data that can be used to address a range of fundamental and applied biological questions (Jones and Good 2016), including medical (e.g., detecting disease variants; Coutelier et al. 2018; Nagy-Szakal et al. 2021) and eco-evolutionary (Jones and Good 2016). In the latter case, the resulting large multi-locus datasets are often used for phylogenomic experiments (Andermann et al. 2020; Ballesteros et al. 2020; Reilly et al. 2022; Zozaya et al. 2022), while the enrichment step makes targeted capture suitable for working with historical and ancient DNA specimens—where the available DNA is present in small amounts and in a highly degraded state (Bi et al. 2013; Derkarabetian et al. 2019; Roycroft et al. 2022).

Depending on the capture design and associated efficiency, significant proportions of the obtained sequence reads may be ‘off-target’, with up to 65% coming from genomic regions that are outside the capture design—e.g., high copy number organellular, such as mitochondrial and chloroplast DNA, bacteria, and viruses (Guo et al. 2012; Samuels et al. 2013). Though usually discarded, such ‘bycatch’ can be leveraged as an important additional source of genomic data, using bioinformatic tools to mine the off-target sequence reads (Guo et al. 2012). For example, Griffin et al. (2014) demonstrated the utility of using off-target exome reads to obtain mitochondrial sequences and identify their pathogenic mutations at a level of accuracy comparable to traditional Sanger sequencing. Assembling mitochondrial sequences from targeted capture bycatch—often to the point of creating complete mitogenomes—is particularly feasible because of the relatively high abundance of mitochondrial DNA (e.g., up to 5% of total sequence reads in human exome sequencing experiments; Gasc et al. 2016). Developments in bioinformatic software have further enabled utilization of bycatch data, for example to detect copy number variation (Kuilman et al. 2015; Laver et al. 2022) from unmapped DNA and RNA reads (Zhang et al. 2016; Gasc et al. 2016; Laine et al. 2019)—including from public data (Vieira and Prosdocimi 2019). Collectively, this work demonstrates the value (and quality; Guo et al. 2012) of sequence data derived from outside targeted regions, and its use for examining a variety of evolutionary questions is growing (e.g., Derkarabetian et al. 2019; Ballesteros et al. 2020; Reilly et al. 2022; Zozaya et al. 2022). However, while the effects of missing data in studies employing phylogenetic inference have been examined (both generally, and in the context of sequence capture; see Tilston Smith et al. 2020, and references therein), its effects on population genetic and phylodynamic analyses—particularly when the data is bycatch and therefore more likely to be patchy in nature—have received less focus.

*Helicoverpa armigera* (the cotton bollworm) is a significant agricultural pest in Asia, Europe, Africa, and Australasia, causing in excess of US$2 billion worth of damage to essential food and fiber crops annually (Tay et al. 2013). High migratory capacity, the ability to feed on a wide range of shared host plants, and rapidly developed resistance to all of the commonly used groups of insecticide chemistry (Fitt 1989; McCaffery 1998; Feng et al. 2005) have facilitated its global spread and impact. *H. armigera* has recently extended its range into South America which, coupled with its potential to also reach North America (Czepak et al. 2013; Tay et al. 2013; Kriticos et al. 2015), poses a serious problem for invasive pest management (Cordeiro et al. 2020; Rios et al. 2022).

As early as the 1960s, taxonomic work described the presence of two subspecies of *H. armigera*—*H. armigera conferta* and *H. armigera armigera*—based on a set of diagnostic wing traits, while phenotypic intermediates between *H. a. armigera* and *H. a. conferta* were reported in the Philippines, Sumatra, and Java (thought at the time to represent the edge of the ‘*H. a. conferta’* range; Hardwick 1965). In 1999, further taxonomic work suggested the presence of ‘Australasian’ and ‘non-Australasian’ populations (i.e., *H. a. conferta* and *H. a. armigera*, respectively; Matthews 1999). Early genetic research focused on resolving population structure generally focused only on local Australian populations (e.g., Endersby et al. 2007; Daly and Gregg 1985; Behere et al. 2007; Song et al. 2015) and used different genetic markers (e.g., allozymes, Daly and Gregg 1985; microsatellites, Daly and Gregg 1985; Endersby et al. 2007; mitochondrial DNA, Daly and Gregg 1985; Behere et al. 2007; Endersby et al. 2007; Anderson et al. 2016; exon-primed intron-crossing (EPIC) markers, Tay et al. 2008; Z-linked EPIC markers, Song et al. 2015, and single-nucleotide polymorphisms (SNPs), Anderson et al. 2016, 2018). Most recently, a combination of mitochondrial and nuclear (SNP) data using Australian samples located in New South Wales (NSW) (Anderson et al. 2018), or NSW and Queensland (QLD) (Anderson et al. 2016) supported the presence of genetically distinct *H. a. conferta* individuals in Australasia, while indicating little population structure (i.e., strong signals of gene flow) among a global panmictic ‘*H. a. armigera’* metapopulation (Behere et al. 2007; Anderson et al. 2016, 2018). However, there has as yet been no comprehensive analysis of population structure in *H. armigera* from widespread and well-sampled locations across Australia, particularly Western Australia (WA), Northern Territory (NT), and Northern QLD.

The *Helicoverpa* system provides an ideal case study for understanding the extent to which targeted bycatch data is suitable for obtaining consistent phylodynamic and population genetic signals because there is an established framework of evolutionary questions that can be examined with a broader geographic dataset*.* Here, we use data from mitochondrial genomes assembled as bycatch from targeted sequence data for historical and contemporary samples collected from across mainland Australia. We examine the effects of missing data on evolutionary inferences, with a particular view toward whether bycatch data can provide consistent conclusions even in the case of high data patchiness (i.e., low-coverage breadth). We further examine how bycatch-derived mitogenome data compares to another source of often publicly available data of varying quality—a region of the mitochondrial cytochrome *c* oxidase gene.

## Materials and methods

### Dataset generation

In McGaughran (2020), a total of 271 pinned specimens of *H. armigera* were obtained from several museums and/or government departments across Australia (including the Australian National Insect Collection (Canberra), the Department of Agriculture and Food (WA), the Department of Agriculture and Fisheries (QLD), the Agricultural Scientific Collections Trust (NSW), and Museum Victoria (VIC)) and used to evaluate the effects of sample age on data quality from targeted sequencing of museum specimens. These samples spanned a range of ages, from 5 to ~ 120 years (McGaughran 2020). We recorded the year and Australian geographic state of collection for 207 of these samples (Supplementary Material Table S1) and combined them with a further 53 samples from Anderson et al. (2016) to examine evolutionary history from the most geographically diverse dataset of Australasian samples to date. Overall, samples in this dataset originated from every Australian state except Tasmania, as well as from Brazil, China, France, India, Madagascar, New Zealand, Senegal, Spain, and Uganda (Table S1).

To obtain mitogenomes as bycatch from Illumina sequencing of the nuclear DNA in McGaughran (2020), we aligned the Illumina sequence reads to the *H. armigera* reference mitogenome (Genbank ID: GU188273.1) using the MEM algorithm of BWA ver. 0.7.5a-r405 (Li and Durbin 2010). Bam files were sorted in samtools ver. 1.5 (Li et al. 2009) and duplicates were removed with picard ver. 2.10.6 (http://broadinstitute.github.io/picard/). Low-quality and ambiguous alignments were removed with samtools commands: -q 20 -f 0 × 0002 -F 0 × 0004 -F 0 × 0008 and bam files were then indexed with samtools. Variants were next identified following the Genome Analysis Toolkit (GATK) ver. 3.8–1 pipeline (McKenna et al. 2010). We used linear regression to determine whether there was a relationship between the proportion of missing mitogenome data and the original sequencing file size (as a proxy for sequencing coverage). To examine the effects of missing data, we subset our bycatch samples into eleven datasets with differing coverage (i.e., proportion of positions for which a base was present) of the reference genome: 5% (*n* = 260), 10% (*n* = 228), 15% (*n* = 204), 20% (*n* = 179), 25% (*n* = 160), 30% (*n* = 145), 35% (*n* = 126), 40% (*n* = 113), 45% (*n* = 105), 50% (*n* = 73), and 65% (*n* = 56).

To provide a complementary analysis to compare our mitogenome results to available published material, we downloaded 817 mitochondrial cytochrome *c* oxidase subunit I (COI) sequences from GenBank (Table S2). These globally distributed *H. a. armigera* COI sequences were combined with our mitogenome data (i.e., total *n* = 1073), aligned using MAFFT ver. 7.408 (Katoh and Standley 2013), and then trimmed, so that the final alignment retained at least 65% coverage of the first 653 bp of the COI gene—resulting in a final dataset of 648 sequences (518 from GenBank). This COI dataset offers further insight into the interplay of dataset composition and coverage, since it represents a high-coverage dataset with a majority of samples labeled as ‘*H. a. armigera*’—the opposite condition to each of the mitogenome datasets, which contain mostly *‘H. a. conferta’*.

### Population genetic analysis

We first conducted a Discriminant Analysis of Principal Components (DAPC) using the adegenet ver. 2.1.2 (Jombart 2008; Jombart et al. 2010; Jombart and Ahmed 2011) package in R ver. 4.3.1 (R Core Team 2017) to explicitly test for the presence of exclusive geographic distributions for distinct *H. a. armigera* and *H. a conferta* genetic clusters. DAPC is a Bayesian approach to clustering samples based on the output of a genomic PCA or prior clustering information. In this case, we had prior clustering information in the form of location of origin of each sample. Thus, for each of the mitogenome and COI datasets, we denoted two clusters (Australia/New Zealand and the rest of the world as ‘*H. a. conferta’* and ‘*H. a. armigera’*, respectively) a priori, allowing the DAPC to reassign samples to each cluster based on a discriminant function analysis. This avoids the need to introduce uncertainty through clustering based on *k-means* analysis of a genomic PCA in the absence of prior clustering information (Jombart and Collins 2022). We took the first 30% of principal components as input for each discriminant factor analysis to avoid inflating probabilities of cluster assignment.

### Phylodynamic analysis

We next performed a phylodynamic analysis of each mitogenome dataset, fitting a Bayesian Coalescent Skyline (BCS; Drummond et al. 2005) to infer demographic history using BEAST ver. 1.10.4 (Suchard et al. 2018). We initially used only Australian samples because coalescent-based skyline methods are sensitive to population structure among data (Ho and Shapiro 2011). We also ran analyses using sampling times only to assess any bias introduced by sampling times in the absence of higher sequence coverage.

In all analyses, we placed an exponential prior with mean 10,000 on the effective population size at the time of the most recent sample, and used a GTR substitution model with four gamma categories and empirical base frequencies. We also placed a gamma prior (shape and scale set to 10 and 10^–7^, respectively) on the substitution rate, corresponding to insect mitochondrial evolution rates in Papadopoulou et al. (2010). All other parameters were left as default and the MCMC chain was run for 2 × 10^8^ steps, with sampling every 10^5^ steps. Using Tracer, we discarded the first 10% of states as burnin, resulting in ESS values above 200 for all parameters (Rambaut et al. 2018). For each dataset, we ran a concurrent analysis with a constant phylogenetic likelihood, which only draws on sampling dates as information (referred to as “sampling from the prior” in BEAST). We used these dates-only analyses to see if the lower coverage sequence data were informative beyond prior configurations and sampling time distributions. From a phylodynamic perspective, using dates-only data is equivalent to analyzing a dataset of 0% coverage samples, which are referred to as *occurrences* in the literature (Featherstone et al. 2021). In this sense, lower coverage samples are informative to some extent between that of an occurrence (i.e., 0% coverage) and a sample with complete genome coverage. It is therefore important to consider the effects of sampling times alone to accurately estimate the value added by low-coverage samples. Dates-only trajectories were omitted in cases where numerical underflow occurred (i.e., when one or more parameter values were too small to be accurately stored and operated on, causing the software to crash).

Finally, we repeated the above mitogenome analyses with the inclusion of non-Australian samples in each dataset. Due to potential population structure in these datasets (see above), we only used them to evaluate support for monophyly (i.e., and not demographic changes) among the *H. a. armigera* samples in the posterior tree distribution of each dataset. We measured this by taking the largest monophyletic *H. a. armigera* clade as a proportion of the total number of *H. a. armigera* samples in a given dataset for each of 1000 subsampled trees from each analysis. A value of 1 thus indicates complete monophyly of the *H. a. armigera* samples and a value of 0 indicates a total lack of monophyly.

### Figure generation

All figures were plotted in R using ggplot2 v3.4.2 (Wickham 2016).

## Results

### Bycatch data quality

**Fig. 1:**
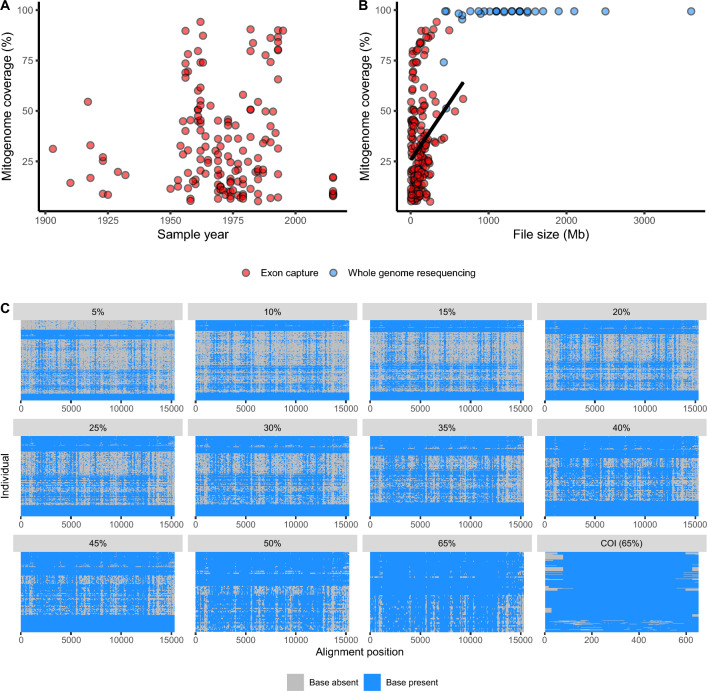
Bycatch coverage results: **a** Proportion of mitogenome coverage versus sampling age; **b** Coverage versus file size; **c** Coverage heatmaps for all mitogenome and the 65% coverage COI datasets. Individuals are represented as rows and are plotted in a random order

Coverage of the mitochondrial genome did not show a clear relationship with sample age (Fig. 1a). However, file size was a weakly positive predictor of coverage (*R*^*2*^ = 0.06; *P* < 0.005 for exon capture data) and generally yielded near-complete coverage for whole genome re-sequencing data from Anderson et al. (2016), for file sizes above 1000 Mb (Fig. 1b,c). This analysis considered file size as a proxy for coverage, but file sizes for other species may provide different results based on changes in the size of the relevant reference genome, among other variables. Across the 5–65% coverage mitogenome and the 65% coverage COI datasets, coverage appeared evenly distributed, despite some small stretches that failed to be captured. The substantial overlap in coverage allowed comparisons between individual mitogenomes, facilitating our subsequent population genetic and phylodynamic analyses.

### Population structure analyses

**Fig. 2:**
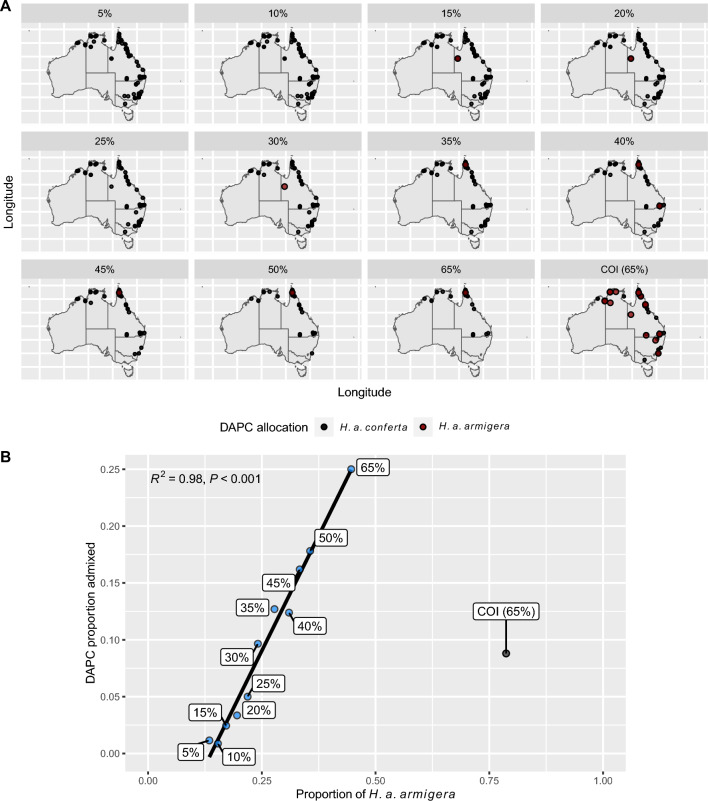
DAPC results: **a** For all coverage mitogenome, and 65% coverage COI, datasets with Australian-collected samples plotted on the map to show the geographic distribution of the DAPC allocations according to the key; **b** The proportion of admixed individuals allocated by DAPC against the proportion of *H. a. armigera* samples in each dataset; points are labeled with the associated dataset and X and Y scales are variable

Our DAPC analyses were used to reassign samples to clusters based on posterior probability assignment (with posterior probabilities in the middle range, here defined as 0.01 to 0.99, indicating admixture between clusters). DAPC results for the mitogenome datasets broadly supported the existence of a distinct Australasian subspecies with minimal admixture among Australasian samples, and site loadings were evenly distributed across the mitogenome (Fig. 2A, Fig. S1). Assignment probabilities fell outside of the admixture interval for the majority of samples, but there was a significant effect of coverage and dataset composition (Fig. 2b). Specifically, the proportion of admixed individuals increased linearly with the proportion of *H. a. armigera* samples in each mitogenome dataset (*R*^*2*^ = 0.98, *P* < 0.001), which itself increased with dataset coverage. Thus, lower coverage affected the robustness of the DAPC to identify admixture (Fig. S2).

The COI dataset contrasted with the mitogenome datasets as it included a higher proportion of *H. a. armigera* samples (25% of the dataset), but returned a comparatively lower signal of admixture (~ 10%) in the DAPC analyses, suggesting that sampling bias alone is insufficient to explain the increased signal for admixture seen in the mitogenome data (Fig. 2b). Instead, higher coverage in the COI dataset appeared to overcome sampling biases and allow for a discriminant function clearly differentiating *H. a. conferta* and *H. a. armigera* samples (Fig. S2).

### Phylodynamic analyses

**Fig. 3:**
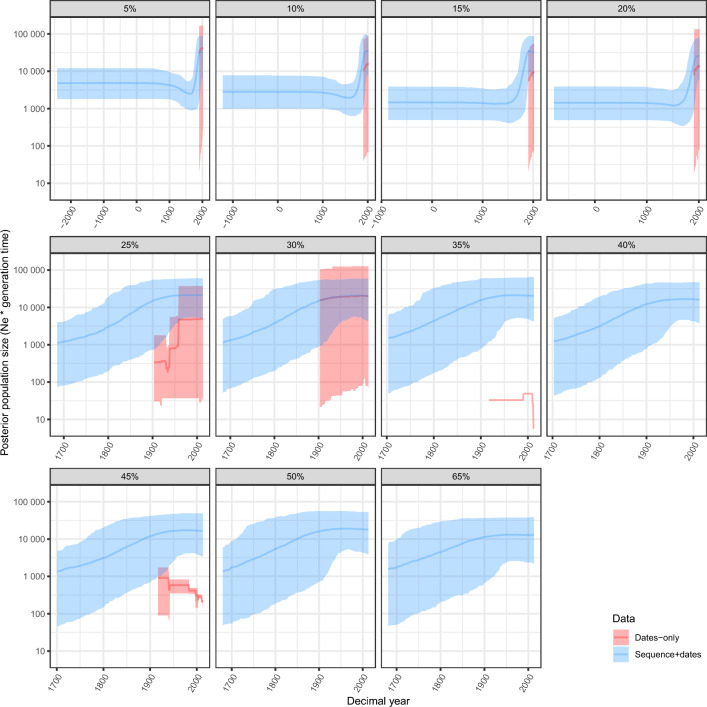
Posterior trajectories of effective population size scaled by generation time (N_e_ * generation time) for all mitogenome coverage datasets. Trajectories are colored by the data type (dates-only or sequence data + sampling times) as indicated by the key. Dates-only trajectories were omitted for datasets where numerical underflow occurred

BCS analyses showed a continual increase in population size from the time of the most recent common ancestor for samples in each dataset, with a plateau from around 1900 (Fig. 3). Datasets including sequences + sampling times yielded different population trajectories to dates-only datasets, affirming that the sequence data were informative in each analysis. However, the posterior population trajectory for the lowest coverage datasets (5–20%) was much older, with a larger burst in population size toward the present than was seen for the datasets with higher coverage (Fig. 3). Thus, less sequence-overlap between individuals in the lowest coverage datasets appears to have driven a signal for an older population trajectory to account for greater diversity among these sequences (in the absence of a higher number of constant sites).

**Fig. 4:**
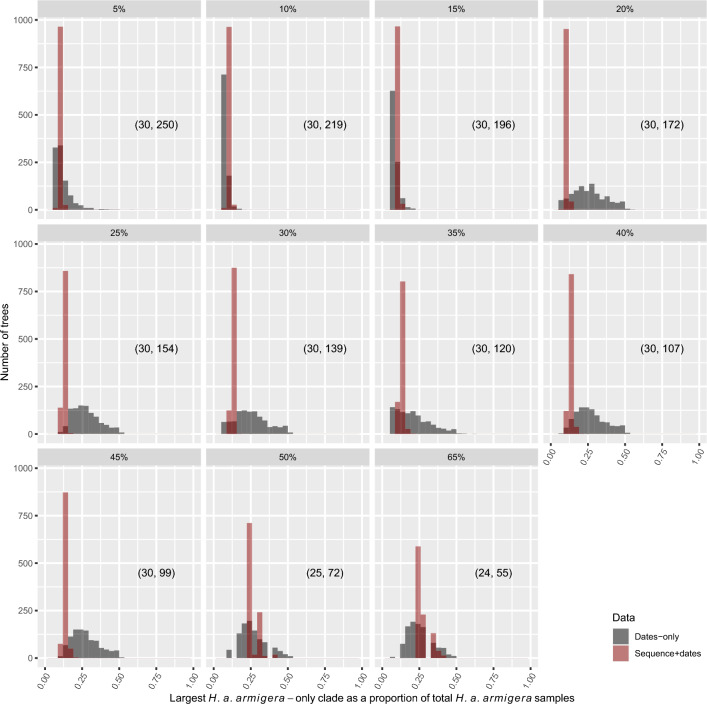
Posterior distributions for the largest *H. a. armigera*-only monophyletic clade as a proportion of the total number of *H. a. armigera* samples. Results are presented for all mitogenome coverage datasets, and for the 65% coverage COI dataset. A value of one indicates that all *H. a. armigera* samples are monophyletic, while 0 indicates no monophyly for *H. a. armigera* samples. Data type (dates: dates-only, seq: sequence data + dates) is indicated by the key. Sequence data + dates trajectories show where analyses are biased in the absence of sequence data. Numbers in parentheses indicate (‘number of *H. a. armigera* samples’, ‘total number of samples’)

To further interrogate the separation between *H. a. armigera* and *H. a. conferta* samples, we re-ran the BCS analyses, including samples from the rest of the world for which sampling times were available. Across all mitogenome datasets, we did not recover any results where *H. a. armigera* samples clustered together as a single monophyletic clade (i.e., we found no posterior support for an *H. a. armigera*-only clade) (Fig. 4). Low proportions (i.e., less monophyly) for dates-only distributions suggested that the sampling time distribution favored less monophyly among *H. a. armigera* samples, and this signal strengthened with the inclusion of sequence data for the 5%-45% coverage datasets. However, the 50% and 65% datasets (which have relatively more *H. a. armigera* samples) showed higher support for monophyly relative to the 5% and 25% datasets.

## Discussion

We aimed to examine the effects of missing data in bycatch obtained from targeted sequencing experiments—using the pest moth, *H. armigera* as a case study to examine these effects in a system with well-considered questions of evolutionary significance. We found that low-coverage sequences broadly supported the delineation of the two *H. armigera* subspecies, with evidence for admixture between the two consistent with previous work. However, we identified important caveats associated with low-coverage bycatch data, as outlined below.

### Bycatch sample quality

Exploring effects of coverage, we found no clear relationship with sample age or the amount of sequencing data obtained per sample (i.e., file size), suggesting that other factors have a stronger effect on the amount and quality of bycatch data that can be obtained. These factors are likely to differ between historical and contemporary species (because the former are highly subject to DNA degradation; Card et al. 2021; Raxworthy and Smith 2021), but sequence variability, repetitive regions and/or paralogy in the target DNA, and hybridization temperature during capture, are all known to affect the amount of off-target reads obtained in targeted sequence experiments (Andermann et al. 2020). The lack of significant age effects affirms that museum specimens—important ‘records’ of historical evolutionary change (Bi et al. 2013; Derkarabetian et al. 2019; Raxworthy and Smith 2021)—offer a rich source of targeted capture bycatch information for species of interest, regardless of their age, at least in the range of sample ages up to ~ 120 years.

### Effects of missing data on evolutionary analyses

Although our tested datasets included up to 95% missing data, our evolutionary results were consistent with each other and the published literature, demonstrating the value of bycatch data to provide or support evolutionary inferences even in the presence of substantially patchy datasets. For example, our mitogenome and COI DAPC analyses supported a clustering pattern corresponding to the *H. a. armigera* and *H. a. conferta* subspecies, with the latter predominating on the Australian mainland. Despite this, we could identify no clear signal of genetic turnover from one subspecies to the other on the Australian continent and our phylodynamic analyses of each mitogenome dataset lacked any support for a monophyletic ‘*H. a. armigera*’ clade. These results are consistent with the most recent previous mitochondrial and genomic analyses of *H. armigera* to include Australasian samples*,* which together indicated an Australasian-specific grouping (i.e., an ‘*H. a. conferta*’ cluster), but also the presence of Australasian samples in the ‘*H. a. armigera*’ cluster and a large degree of admixture between *H. a. armigera* and *H. a. conferta* samples at the genomic level (Anderson et al. 2016, 2018).

Two potential explanations for these evolutionary patterns are: (i) that the subspecies are not geographically exclusive, but co-exist across at least some sites in Australasia and perhaps other locations in the world; or (ii) that the subspecies are geographically exclusive, but sex-biased dispersal, selection, demographic events, (re-)introduction of *H. a. armigera* into Australia through admixture, or some combination of these, has led to the observed patterns (Després 2019). These questions are beyond the scope of the current research, where our intent was to explore the effects of missing data in bycatch analyses, however they should be examined further with genomic data that includes a wider range and number of Australasian samples. Of particular interest, admixture between Australasian and Chinese samples (Anderson et al. 2016, 2018), coupled with the recent population growth of *H. a. conferta* identified in our BCS analyses, suggest the potential for a region of turnover between subspecies proximal to mainland Asia. Investigating this further may be important for pest management efforts, particularly if subspecies status (versus population distinctiveness given that nuclear measures of genetic differentiation between *H. a. armigera* and *H. a. conferta* are extremely low: F_ST_ < 0.001; Anderson et al. 2018), bears significance for management of this global pest species.

Despite the general consistency of our evolutionary results with published work, we found that samples with different coverage thresholds presented different specific findings. For example, while the degree of identified admixture increased (i.e., the support for discrete subspecies clusters decreased) with increasing mitogenome dataset coverage and proportion of *H. a. armigera* samples, no such pattern was apparent in the COI dataset for the DAPC analyses. In our BCS analyses, inferences of population size differed with mitogenome coverage—especially the 5–20% datasets—while the 50% and 65% mitogenome datasets (with more *H. a. armigera* samples) showed higher support for monophyly relative to the lower coverage datasets. This suggests that comparison of samples with different coverage thresholds is critical for separating the effects of sampling and coverage biases from genuine genomic signals, particularly when coverage is < 25%. Thus, while low-coverage bycatch data can offer valuable information for population genetic and phylodynamic analyses, users should quantify the degree of missing data in their bycatch to best understand its implications for phylodynamic and high-dimensional approaches, such as DAPC.

The presence of missing data prevents applicability of some population genetic and/or phylogenetic analyses. For example, haplotype networks can provide spurious results in the presence of missing data (Joly et al. 2007; Carreras et al. 2014). Meanwhile, temporal data (e.g., from museum specimens) may not meet standard phylogenetic assumptions of isochronous sampling, requiring the use of more highly parameterized phylogenetic analyses (Rieux and Balloux 2016). Nevertheless, the additional data obtained from bycatch allows for a wider scope of research and greater potential insights into an array of applications. For example, in previous human research, high-quality SNPs from outside target regions bolstered tested datasets by up to 461% (Guo et al. 2012). Indeed, this is a growing field (e.g., Derkarabetian et al. 2019; Ballesteros et al. 2020; Granados Mendoza et al. 2020; Sanderson et al. 2020; Costa et al. 2021; Reilly et al. 2022; Zozaya et al. 2022), and we recommend that more researchers consider the extraction and analysis of bycatch data (as well as other off-target genomic resources, such as unmapped RNA reads in transcriptomic studies), in their informatics pipelines. Although some of these data will undoubtedly represent contamination and/or poor quality sequences, what remains may provide the raw material for new avenues of active research (Samuels et al. 2013; Griffin et al. 2014; Seaby et al. 2016). This will be particularly relevant if researchers have access to a suitable reference genome against which to align their sequence reads and/or lack any available mitochondrial or nuclear population genetic data for their target species, as well as for co-evolution or adaptive introgression (i.e., mito-nuclear discordance) research.

### Supplementary Information

Below is the link to the electronic supplementary material.

Supplementary file1 (DOCX 233 KB)

Supplementary file2 (CSV 14 KB)

Supplementary file3 (CSV 16 KB)

## Data Availability

We provide full sample details in Supplementary Material Tables S1 and S2. The FASTA data files used in our analyses, all scripts for DAPC and phylodynamic analyses, and scripts for making figures are available at: https://github.com/LeoFeatherstone/helicoBycatch.
