# Supplementary material for: The effect of missing data on evolutionary analysis of sequence capture bycatch, with application to an agricultural pest
Source: Mol Genet Genomics. 2024 Feb 21;299(1):11. doi: 10.1007/s00438-024-02097-7 (PMC10881687; doi:10.1007/s00438-024-02097-7)
Supplement: Supplementary file 1 — Supplementary file1 (DOCX 233 KB) [file 438_2024_2097_MOESM1_ESM.docx]

**Supplementary Material**

**The effect of missing data on evolutionary analysis of sequence capture bycatch, with application to an agricultural pest**

Leo A Featherstone^1,2^, Angela McGaughran^1,3,*^

^1^Research School of Biology, Division of Ecology and Evolution, Australian National University, Canberra, ACT 2601, Australia

^2^Peter Doherty Institute for Infection and Immunity, The University of Melbourne, Melbourne, VIC 3000 Australia

^3^Te Aka Mātuatua - School of Science, University of Waikato, Private Bag 3105, Hamilton 3240, New Zealand

**Corresponding Author:** Angela McGaughran; amcgaugh@waikato.ac.nz

**Figure S1.** DAPC loadings for each nucleotide site across each all mitogenome and the COI datasets.

**
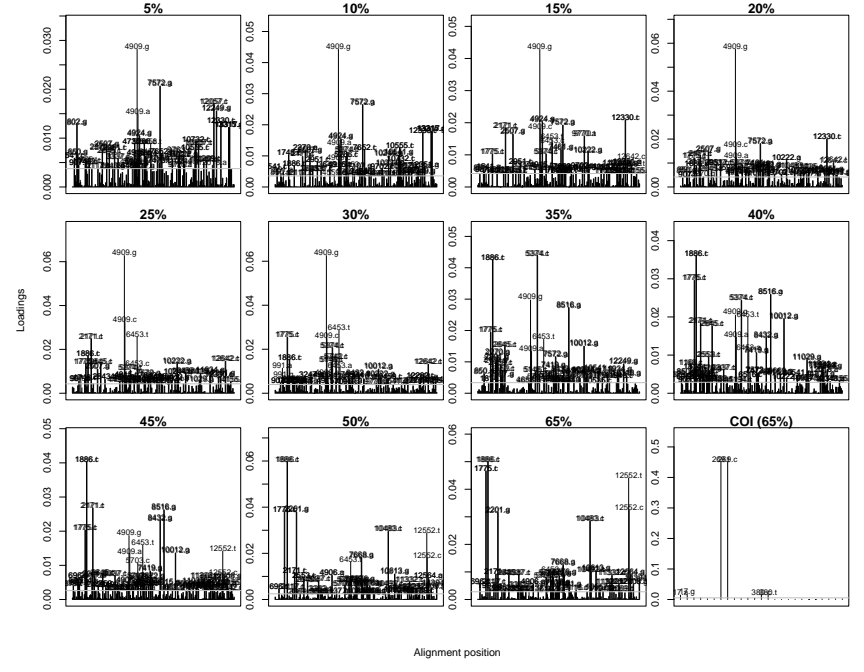
**

**Figure S2.** Histograms of DAPC discriminant function values for each sample in each mitogenome and COI dataset, coloured by allocated DAPC cluster, as indicated by the key. Note that X and Y scales are variable.


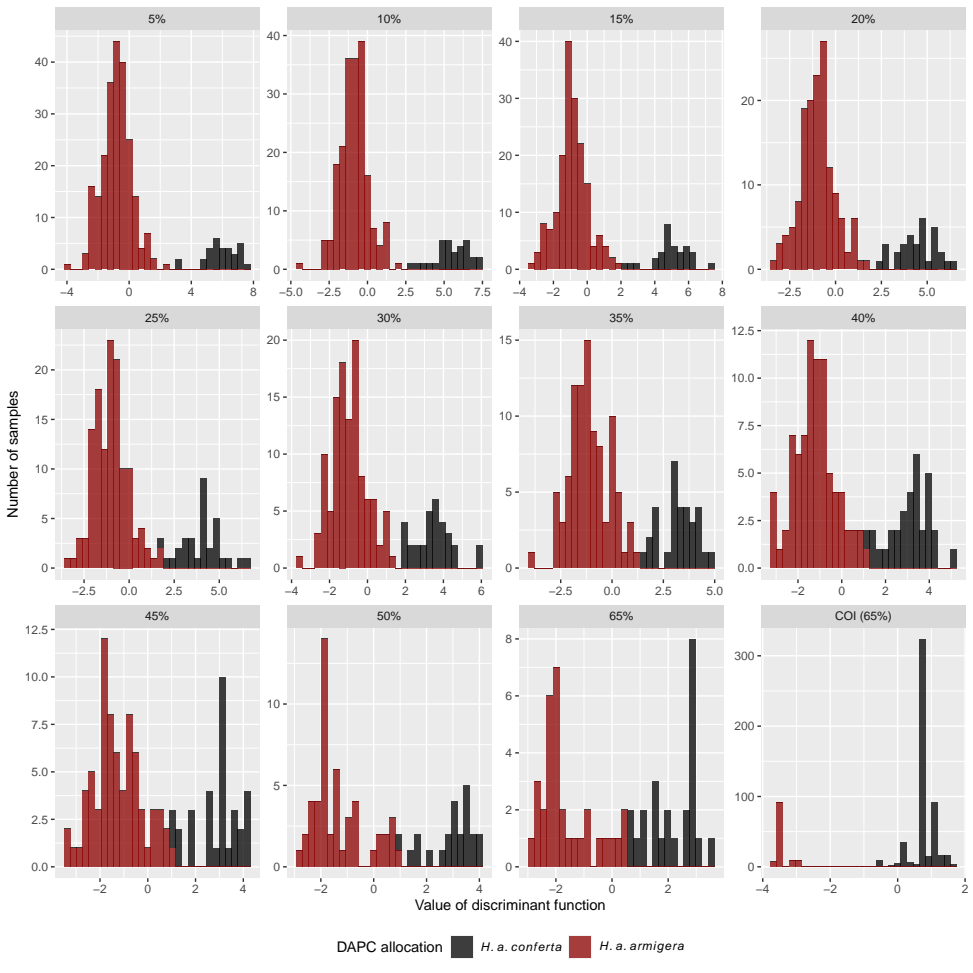


**Table S1** List of all individual samples from McGaughran (2020) and Anderson et al. (2016) used in the present study, with sample ID, country of origin, state, locality, year of collection, latitude, and longitude indicated.

**Table S2** List of individual samples downloaded from GenBank and used in COI analyses, with sample ID, GenBank accession number, and 3-letter country code indicated.
